# Multiple sclerosis and exercise—A disease-modifying intervention of mice or men?

**DOI:** 10.3389/fneur.2023.1190208

**Published:** 2023-10-10

**Authors:** Sarah-Jane Martin, Raphael Schneider

**Affiliations:** ^1^BARLO MS Center, St. Michael's Hospital, Toronto, ON, Canada; ^2^Keenan Research Centre for Biomedical Science, St. Michael's Hospital, Toronto, ON, Canada; ^3^Institute of Infection & Immunity, University of Glasgow, Glasgow, Scotland, United Kingdom; ^4^Institute of Medical Science, Temerty Faculty of Medicine, University of Toronto, Toronto, ON, Canada

**Keywords:** multiple sclerosis, exercise, experimental autoimmune encephalomyelitis, translational research, biomarkers

## Abstract

Research suggests that physical exercise can promote an anti-inflammatory and neuroprotective state. If so, increasing or optimizing exercise could be considered a 'disease-modifying intervention' in neuroinflammatory diseases, such as multiple sclerosis (MS). Exercise intervention studies conducted in animal models of MS are promising. Various aerobic and strength training regimes have been shown to delay disease onset and to reduce both the clinical and pathological disease severity in mice. However, fundamental differences between the physiology of animals and humans, the disease states studied, and the timing of exercise intervention are significant. In animal models of MS, most exercise interventions begin before disease initiation and before any clinical sign of disease. In contrast, studies in humans recruit participants on average nearly a decade after diagnosis and often once disability is established. If, as is thought to be the case for disease-modifying treatments, the immunomodulatory effect of exercise decreases with advancing disease duration, current studies may therefore fail to detect the true disease-modifying potential. Clinical studies in early disease cohorts are needed to determine the role of exercise as a disease-modifying intervention for people with MS.

## Introduction

Multiple sclerosis (MS) is an immune-mediated disorder of the central nervous system (CNS). The underlying pathological process is a triad of inflammation, demyelination, and neurodegeneration, with inflammation particularly dominant in early disease (1). Clinically, MS can be described as 'relapsing remitting MS' (RRMS), where acute bouts of neurological deficit are followed by variable recovery and stability, or as progressive MS [primary progressive (PPMS) or secondary progressive (SPMS)], where insidious accumulation of disability occurs with less pronounced relapses (2). Highly effective disease-modifying treatments (DMTs) that target immune cells have revolutionized the management of RRMS (3, 4). However, despite significantly reducing clinical and radiological evidence of focal disease activity, the success of DMTs in preventing disease progression has been relatively disappointing. Progression independent of relapse activity still occurs (5). Prescription of DMTs in progressive MS remains restricted and their use may also be limited over concerns of their risk of potentially serious adverse effects. This is particularly true *early* in the disease course when patients are often young with minimal disability. Unfortunately, it is at this point, early in the disease, when DMTs have the greatest potential to impact the long-term risk of disability (4).

## Exercise as a disease-modifying intervention

A “disease-modifying intervention” (DMI) describes the act of altering a modifiable trait or state with the aim of delaying, slowing or lessening disease progression. In MS, DMIs might include smoking cessation (6), reducing obesity (7), or changes to diet (8). DMIs are a separate entity from disease-modifying treatments and should be considered in *all* patients with MS (pwMS), irrespective of DMT status.

Exercise is defined as a purposeful, effortful physical activity performed to promote or maintain health or fitness. Exercise can take numerous forms but can be broadly characterized as resistance training or aerobic activity. Resistance training strains the neuromuscular system, boosting anaerobic endurance, increasing muscle strength, and enhancing bone health (9). Aerobic exercise taxes the cardiovascular system, increasing aerobic capacity (9). Aerobic capacity is a strong health predictor and is associated with lower all-cause mortality (10).

Research conducted in animal models of MS suggests that exercise may modulate the systemic immune system to promote an anti-inflammatory and neuroprotective state within the CNS (11). If so, exercise might also be considered a DMI (12, 13). However, whether the immunomodulatory effects of exercise in rodents translate to humans, and, if so, how such effects might be maximized in the care of pwMS, is unknown.

## Methods

To better understand whether the disease modifying effects of exercise seen in animal models of MS are potentially clinically translatable, we conducted a literature review comparing preclinical and clinical exercise intervention studies. As this was a literature review, no priori review protocol or search strategy were implemented. Based on our findings, we then conduced systematic reviews of the timing of exercise intervention in the most commonly employed animal model of MS (experimental autoimmune encephalomyelitis – EAE) and in people with MS. We developed illustrative figures based on these results. The search strategies are depicted in Table 1. All statistics were calculated using GraphPad Prism version 8.4.3. Data were not normally distributed and are depicted as median and interquartile range. Figures were created using GraphPad prism and BioRender.com.

**Table 1:** Systematic search strategy to determine the timing of exercise interventions in animal models of MS compared with clinical studies in MS cohorts.

**The timing of exercise intervention studies in animal models of MS**	
Date	January 13, 2023
Search strategy Pubmed (Title/Abstract)	“Experimental autoimmune encephalomyelitis” AND “exercise” AND “mice”
Total hits	23
Abstracts reviewed and excluded	2
Full text reviewed and excluded	1
Included in final analysis	**20** (see Supplementary Table 1)
Inclusion/exclusion criteria	Studies were restricted to the EAE rodent models of MS (19 mouse and 1 rat). Both active immunization and passive transfer EAE models were included. Studies of mechanisms and actions of drugs were excluded. Studies assessing the impact of exercise on pain were excluded. Exercise regimes could be voluntary (enriched environment) or forced, and could include running, climbing, swimming etc.
The timing of exercise intervention studies in clinical studies in MS cohorts	
Date	January 9, 2023
Search strategy: Pubmed (Title/Abstract)	“*Meta-analysis” AND “Multiple sclerosis” AND “exercise”*
Total hits	81
Abstracts reviewed and excluded	52
Full text reviewed and excluded	18
Included in final analysis	**11** (see Supplementary Table 2)
Inclusion/exclusion criteria	Studies were excluded if the exercise intervention was vestibular rehabilitation, respiratory muscle training, sexual function rehabilitation or massage therapy. Studies that measured purely psychiatric outcomes, autonomic reflexes, flexibility, pain measures, standing time, falls risk or spasticity measures were excluded. Studies that involved a component of inpatient rehabilitation were also excluded. Exercise regimes could include any activity that was deemed to have an active component, such as home or group-led circuit training, aerobic or resistance/strength training, swimming, sports climbing, robot-assisted training, virtual reality and video-game-led exercise (Wii etc), yoga or Pilates. Most meta-analyses analyzed the impact of the exercise intervention on more than one outcome, for example, “the effect of exercise on lower limb physical function and perceived fatigue.” In such cases, studies were included only once, in whichever was thought to be the primary outcome.

## Exercise intervention studies are conducted too early in animal models of MS

Animal models of MS enable exercise intervention studies to be conducted over short periods of time, at clearly defined points in the disease, and to link clinical outcomes with pathological analysis; and results are promising. Both aerobic and resistance training regimes have been shown to delay disease onset and reduce both clinical and pathological disease severity (14–18). These outcomes are thought to primarily arise through modulation of the systemic immune system; however, the specific mechanism of modulation appears to differ depending on the type and intensity of exercise.

Experimental autoimmune encephalomyelitis (EAE) is the most employed animal model of MS. EAE can be induced by immunization with myelin proteins (usually myelin oligodendrocyte glycoprotein, MOG, or proteolipid protein, PLP) or through adoptive transfer of autoreactive T cells. Different models result in different EAE phenotypes. For example, PLP-induced EAE is reported to be milder, with a remitting-relapsing course, compared with the chronic disease course of MOG-induced EAE (19). Spontaneous EAE models also exist. In chronic EAE models (where mice develop a chronic motor deficit), strength training has been shown to upregulate peripherally circulating regulatory T cells (Tregs), whereas endurance training had a greater effect on restoration of blood-brain-barrier (BBB) integrity (15). High-intensity interval training has been shown to reduce populations of pro-inflammatory T helper (Th) cells (Th1 and Th17) specifically, whereas high-intensity continuous training led to a more general reduction in T cell populations (20). Studies in different animal models, such as the Cuprizone and lysolecithin-induced demyelination models, suggest that exercise may also exert effects directly within the CNS through limitation of microglial activation (18) and increase of local anti-oxidant responses (21).

**Figure 1:**
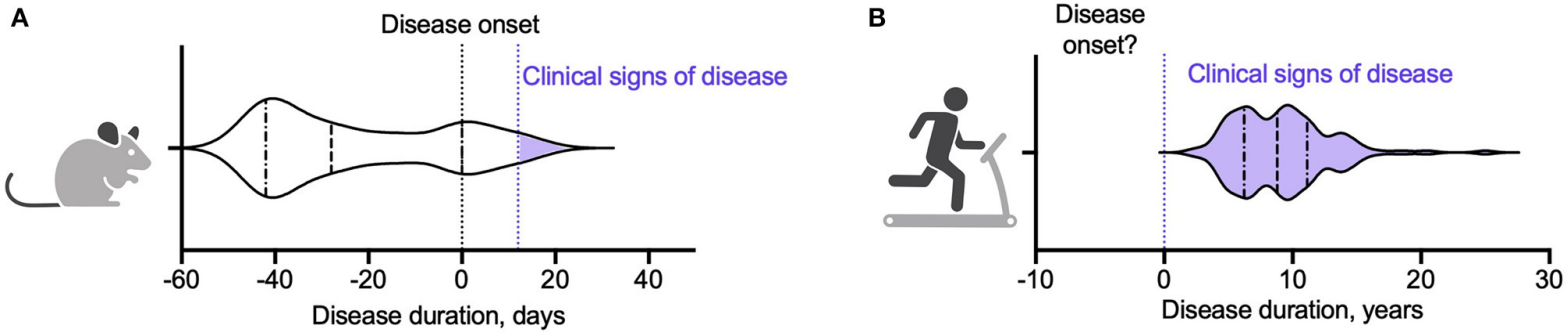
Violin plots showing point of initiation of exercise intervention in **(A)** an animal model of MS and **(B)** people with MS. **(A)** Summary of twenty studies assessing the impact of exercise on clinical, immunological, and pathological outcomes in EAE (15–17, 20, 23, 53–67). Animals began training a median of 28 days prior to EAE induction (denoted as day 0). The inter-quartile range was −42 to 0 days. Fourteen studies (70%) initiated and/or completed the exercise intervention prior to EAE induction. One study initiated the exercise intervention on day 0 (63). Of the five studies that started the exercise intervention after induction of EAE, three began the exercise programme on day 1 (prior to the animals developing clinical signs of disease) (53, 61, 62) and two had subgroups that began exercise at the point the animals developed clinical signs of disease (day 12 and day 18) (16, 23). **(B)** Summary of 117 studies from eleven meta-analyses assessing the impact of exercise interventions on clinical and immunological measures in pwMS (30, 31, 34, 35, 50, 51, 68–72). Study participants had a median disease duration of 8.8 years. The inter-quartile range was 6.2–11 years.

There are of course limitations in drawing conclusions from animal models. Animal models only partly mimic the pathology of MS; rodent stamina, activity levels, and innate drive to exercise differ significantly from humans; and certain training regimes, such as forced exercise, cannot be recapitulated and the effect of stress may independently alter the disease course (22). In addition, we noted that most studies in animal models begin the training regime *prior to initiation of the disease state*, Figure 1 and Supplementary Table 1.

Few studies have examined the effects of an exercise intervention delivered *after* induction of EAE, and those that have report mixed results. Klaren et al. (23) found no significant effect of exercise intervention on clinical disability scores when animals exercised during remission after initial disease onset in a PLP-induced relapsing remitting EAE model. In contrast, Le Page et al. (14) found that 2 days of severe exercise after transfer of encephalitogenic T cells delayed disease onset (but not ultimate disease severity), whereas the same exercise regime prior to induction of adoptive EAE did not change the disease course (14). Finally, Shahidi et al. (16). showed that aerobic exercise reduced clinical and pathological severity of EAE equally, regardless of whether mice started training before EAE induction or after the clinical onset of disease. Variability in results from these studies may reflect the relatively short window (two-three weeks) between disease initiation and animal sacrifice. Beginning an exercise intervention only after initiation of disease reduces the duration of training (and potentially also the training intensity), and as such may underestimate potential beneficial effects (22).

## Exercise intervention studies are conducted late in people with MS

In healthy humans, moderate-intensity exercise (in particular) has been shown to promote an anti-inflammatory and neuroprotective state through multiple pathways (24). Myocyte release of interleukin-6 (IL-6) upregulates the release of anti-inflammatory cytokines (such as IL-10 and IL-1 receptor alpha) and downregulates the release of pro-inflammatory cytokines (such as TNF-α and IFN-γ) (24–26). Proliferation of naive cell subsets alongside apoptosis of quiescent and exhausted cells alters circulating cell profiles (24, 26). Exercise has also been shown to enhance expression of neurotrophins, such as brain derived neurotrophic factor (BDNF), that regulate neurogenesis, neuronal function and survival (27). Finally, exercise may also modulate glia cell phenotype and function (28).

However, whether biological and physiological responses to exercise differ in the context of immune dysregulation is not clear. For example, one meta-analysis found that, in contrast with controls, neither acute nor regular exercise led to a significant change in peripherally circulating IL-6 in pwMS (29). Moreover, whether the immunomodulatory changes that occur in the context of exercise might have a *clinically meaningful* impact on the disease course in MS requires specific studies.

**Figure 2:**
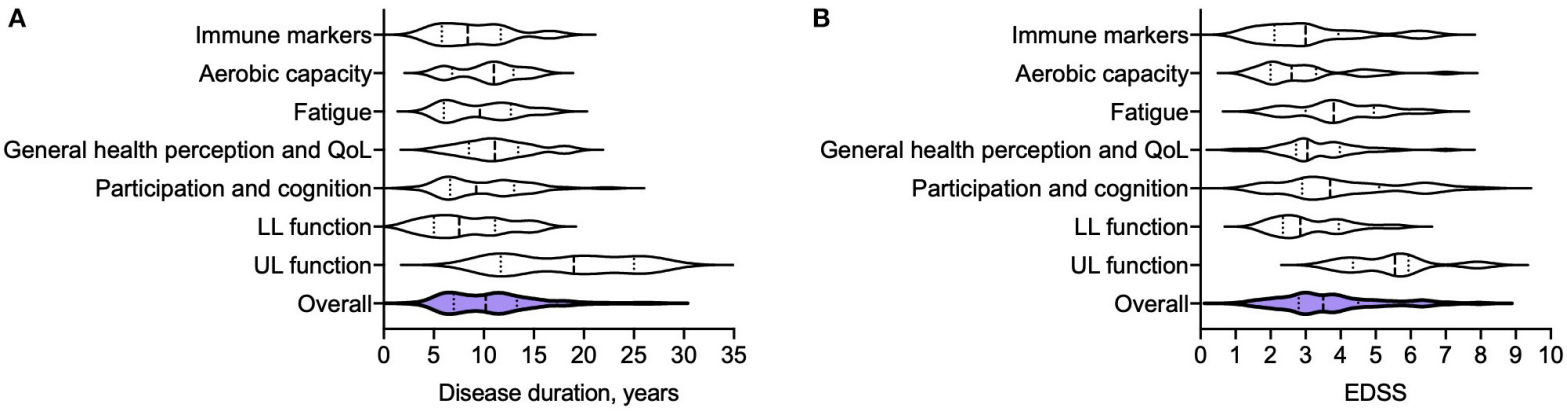
Disease duration **(A)** and EDSS **(B)** of MS participants from 166 different exercise intervention studies that were summarized in 11 meta-analyses (30, 31, 34, 35, 50, 51, 68–72). Disease duration and EDSS are reported as median (years), and interquartile range. Immune markers: Disease duration 8.4 (5.8–12), EDSS 3 (2.1–3.9) (35, 72); Aerobic capacity: Disease duration 11 (6.8–13), EDSS 2.6 (2–3.3) (31); Fatigue: Disease duration 9.6 (6–13), EDSS 3.8 (3–5) (50); General health perception and quality of life (QoL): Disease duration 11 (8.5–13), EDSS 3.1 (2.7–4) (34, 51, 70);Participation and cognition: Disease duration 19 (12–25), EDSS 5.6 (4.5–5.9) (68, 71); Lower limb function: Disease duration 8.1 (5–11), EDSS 2.9 (2.4–4) (69); Upper limb function: Disease duration 9.3 (6.6–13), EDSS, 3.7 (2.9–5.1) (30); Summary: Disease duration of overall cohort (from 117 separate studies) 8.8 (6.2–11), EDSS of overall cohort (from 166 separate studies) 2.7 (2–3.5).

The literature on exercise intervention studies in pwMS is expansive, with huge heterogeneity in participant cohorts, training regimes, study durations and endpoints. It is notable however that the majority of studies are conducted in MS cohorts with long disease durations and established disability, Figure 2, Supplementary Table 2. This is perhaps unsurprising, given the traditional view of exercise as rehabilitation (13). For example, studies of upper limb strength training tend to recruit individuals with arm weakness or for whom upper limb function is particularly important, such as wheelchair users. Such cohorts are likely to have long disease durations and high disability scores [measured by the Expanded Disability Status Scale (EDSS)], as demonstrated in a systematic review by Neira et al. (30) in which participants across eight studies of upper limb strength training had average disease durations between 9 and 27 years, and EDSS scores between 3.5 and 9.

Exercise intervention studies of lower limb function or aerobic capacity may have lower average EDSS scores, but are often heterogeneous. Langeskov-Christensen et al. (31) analyzed the effects of aerobic training on aerobic capacity across 17 randomized control trials of participants with an EDSS range of 0 to 8. A significant effect in favor of exercise intervention was found, but heterogeneity between results was thought to reflect variability between study cohorts. One potential explanation posed was that deconditioning in higher EDSS groups may limit the degree of improvement in aerobic capacity over a short study period (31).

Studies across the entire clinical spectrum of MS are needed to understand the specific effects of exercise at different disease stages. However, if, as is thought to be the case for DMTs, the immunomodulatory potential is greatest in early disease, current exercise studies may miss the “window of opportunity” in which to exert a clinically meaningful effect on the disease course (4, 32). To date, few clinical studies have been conducted specifically in *early disease cohorts* (32).

## Can evidence from animal studies inform the design of exercise studies in pwMS?

Animal models provide important mechanistic insights. For example, results suggest that various types of exercise have the potential to induce immunomodulatory effects, and that future exercise intervention studies might not need to restrict participants to a specific modality or regime. Preclinical studies have provided valuable insights into the anti-inflammatory effects associated with exercise. Although few preclinical studies have investigated the effect of exercise intervention *after* disease onset, those that have suggest the role of exercise as DMI is worth pursuing in clinical studies (16).

Human studies, in “real-world” cohorts (and ideally in early disease) are required to learn how to optimize exercise regimes in order to maximize therapeutic effects. Real-world clinical studies are also required to identify measures or biomarkers that could be employed in the monitoring of an exercise activity or the response to exercise, and then linked with long-term clinical outcomes.

Wearable biosensor technologies could be employed to monitor exercise adherence and provide an estimate of exercise intensity (33). Depending on the exercise intervention, quantification of VO2max (31), “1 rep max” strength (34), grip strength (30) or timed walk test could be used as a measure of the overall physical effects of exercise. The identification of a specific fluid biomarker that might best reflect an individual's immune response to exercise is more difficult. Changes in circulating cell profiles are found pre-clinic studies, but results are heterogeneous, and analysis expensive to perform and labor intensive. Protein biomarkers may be most easily adapted to clinic studies (and thus clinical practice).

BDNF is a member of the neurotrophin family. Neurotrophins play an essential role in neuro-regeneration and neuroprotection (27, 35). In pwMS, blood BDNF levels have been reported to increase during relapse (potentially as a compensatory mechanism) and normalize during remission (35). A recent meta-analysis of 13 studies found that physical activity in pwMS significantly increased baseline serum BDNF levels (35), as has been previously described in healthy populations. However, when broken down into subgroups, no significant differences were identified related to exercise type or duration. Although the study was unable to identify a direct connection between exercise intensity or duration with BDNF alterations, it is possible that variations in study designs, the small size of study groups, diverse patient backgrounds, and different BDNF measurement techniques might obscure the potential effects of specific exercises and their duration on BDNF levels.

IL-6 is a pleotropic cytokine that has been shown to have both pro and anti-inflammatory effects, possibly depending on the source of the cytokine (29). IL6 secreted by B cells, T cells and macrophages, induces synthesis of acute phase proteins, and stimulates antibody production and effector T cell development (36). IL-6 is upregulated in myocytes during exercise in response to a fall in muscle glycogen content and in proportion to exercise intensity (24). IL-6 released from myocytes may upregulate the release of anti-inflammatory cytokines (such as IL-10 and IL-1 receptor alpha) and downregulate the release of pro-inflammatory cytokines (such as TNF and IFN-γ) (24–26, 37).

IL-6 has been studied as a biomarker of the immune effects of exercise in athletes, controls and disease populations (24). Studies in pwMS show inconsistent results. Some report an increase in IL-6 in both pwMS (RRMS) and controls (38), whereas, others reporting no significant acute or long-term change in IL-6 in pwMS (progressive MS) following training (39). One potential reason for discrepancy may be variability in the exercise regimes or intensity achieved between RRMS and progressive MS populations.

TNF is another pleiotropic cytokine that is thought to exert both pro- and inti-inflammatory effects and play a role in immune dysregulation, neuroinflammation and demyelination in MS (40). In post-mortem studies, high TNF levels are found in close proximity of MS lesions (40). Several studies have demonstrated elevated TNF levels in blood or CSF, with some showing a correlation with disease progression or disease activity (29, 38, 40).

In healthy controls, TNF concentrations have been shown to increase immediately following resistance training and recover within 24 h (41). Similar responses have been reported in pwMS (38). Interestingly, one study found that whilst resting levels of cytokines (including TNF) did not differ between sedentary and trained pwMS, sedentary pwMS show a blunted cytokine response to exercise compared with trained pwMS (42). However, in contrast, meta-analysis of 11 studies reported that baseline TNF levels in the blood of pwMS decreased after regular exercise (29), suggesting that regular exercise may have an anti-inflammatory effect on blood TNF levels in pwMS.

Protein biomarkers of axonal damage, such as neurofilament light chain (NfL), or astroglial activation, such as glial fibrillary acidic protein (GFAP) may serve as ‘endpoint' biomarkers. In one randomized control trial (RCT), 38 individuals with RRMS who participated in an 8-week aerobic training programme had significantly lower serum NfL and GFAP levels post-training compared with pre-training. No significant changes in either biomarker were observed in the control group (43). However, another RCT of 89 pwMS found no significant change in either NfL or GFAP over a 16-week aerobic training period (44).

Whilst there are many potential confounders (including age; sex; genetics; co-morbidities; concurrent infection; type, duration and effort of exercise; and timing of blood-draw in relation to exercise), the identification of biomarkers that provide insight into an individual's immunomodulatory responsiveness to exercise could enable “personalized prescription” of exercise as a component of precision medicine (45). This would be a significant shift from the current status quo where exercise is viewed as a tertiary therapy for rehabilitation, to exercise as a therapy for secondary (13, 32), or possibly even primary prevention (13, 46, 47).

Human studies cannot replicate preclinical models in initiating an exercise intervention prior to clinical disease onset. However, several retrospective analyses of physical activity and risk of MS have been conducted to study the potential primary preventative effects of exercise in humans.

A large case-control study across several European countries found that vigorous physical activity in adolescence was inversely associated with risk of MS, with an odds ratio of 0.74 after adjusting for confounders such as outdoor activity, body size, smoking and infective mononucleosis (48). Another study analyzed a historical cohort who undertook mandatory conscription at age 19 years and found a similar inverse relationship between aerobic fitness and MS risk, with an adjusted relative risk of 0.69 (46). Directionality of causality is always a consideration, however results remained significant when men who developed MS within 10 years of conscriptions were excluded from analysis (46). These studies raise the question whether greater physical fitness truly protects against developing MS, or, as is seen in animal models, postpones clinical presentation (13, 47); and if so, whether postponement occurs because the disease course has been modified, or simply because a greater physical reserve might better mask disability.

## Promoting exercise as routine clinical care for all pwMS

Exercise has a low-risk profile and is safe for pwMS. In addition to the physical gains in endurance, balance and strength, positive effects on fatigue and mood can improve quality of life and work outcomes (49–52). This is significant as symptoms such as fatigue are common, intrusive, and difficult to treat; and DMTs have little impact. For these reasons, the *general health* benefits of exercise should be promoted to all pwMS, but further research is required before we can advocate the *disease modifying* effects.

## Conclusion

Research conducted in animal models suggests that exercise has the potential to modify the disease course of MS, particularly if prescribed early and alongside DMTs (11, 17). However, the substantial discord between preclinical research and “real-world” clinical studies limits our ability to determine whether exercise regimes that produce immunomodulatory effects in animals are feasible, practical, and clinically translatable. Clinical studies, specifically in early disease cohorts, are needed to determine whether exercise could have a role as a disease modifying intervention for people with MS.

## Author contributions

S-JM: data curation, analysis, and visualization. All authors contributed equally to conceptualization, literature review, manuscript reviewing, and editing.
