# Supplementary material for: Multiple sclerosis and exercise—A disease-modifying intervention of mice or men?
Source: Front Neurol. 2023 Oct 10;14:1190208. doi: 10.3389/fneur.2023.1190208 (PMC10598461; doi:10.3389/fneur.2023.1190208)
Supplement: Supplementary file 1 [file Table_1.docx]

**Supplementary Table 1** outlining twenty exercise-intervention studies conducted in EAE animal models and the timing of the exercise intervention in relation to disease initiation

| Study | Animal model | Training regime | Timing of initiation of exercise intervention with respect to induction of EAE | Outcomes |
| --- | --- | --- | --- | --- |
| *Rossi S*  *et al 2009* | MOG-induced chronic EAE model. Female C57BL/6 mice were immunized with MOG_35-55_ peptide in CFA supplemented with *M.Tuberculosis*. N=36 | Voluntary exercise vs Sedentary  Exercise group were housed with a running wheel. | Wheel access initiated from day of immunization (day 0). | Motor disturbances in exercising animals were attenuated in both acute and chronic stages of EAE. |
| *Bernardes D et al 2013* | MOG-induced chronic EAE model. Female C57BL/6 mice were immunized with MOG_35-55_ peptide in CFA supplemented with *M.Tuberculosis.* N=67. | Forced exercise vs Sedentary  Forced exercise consisted of 30 minutes swimming per day, 5 days/week for 6 weeks with 7% body weight attached to tail. | EAE was induced following 4 weeks of training (day -28). | Trained animals had significantly attenuated disease severity scores and body weight loss from days 10-14 dpi, at which point animals were sacrificed. |
| *Benson C*  *et al 2015* | MOG-induced chronic EAE model. Female C57BL/6 mice were immunized with MOG_35-55_ peptide in CFA supplemented with *M.Tuberculosis*. N=60. | Voluntary exercise vs Sedentary  Exercise group had voluntary wheel access for 1 hour/day. | Wheel access initiated 1 day after immunization (day +1). | Animals in the voluntary exercise group had a delayed disease onset. |
| *Pryor W*  *et al*  *2015* | MOG-induced chronic EAE model. Male C57BL/6J mice were immunized with MOG_35-55_ peptide in CFA supplemented with *M.Tuberculosis*. N=8. | Voluntary exercise vs Sedentary  Animals were housed with a running wheel or locked wheel. | Wheel access initiated 1 day after immunization (day +1). | Animals in exercise group had a significant delay in development of EAE clinical grade 1 and lower mean disease scores up to day of euthanasia. |
| *Klaren R*  *et al 2016* | PLP-induced RR-EAE model. Female SJL mice were immunized with PLP_139-151_ in CFA supplemented *M.Tuberculosis.* N=47. | Voluntary vs Forced exercise vs Sedentary  Animals were housed with a running wheel (voluntary exercise) or locked wheel (sedentary). Forced exercise consisted of 30 minutes treadmill running, 5 days/week. | Exercise regimes were delivered during remission (approximately 18 days following immunization) and continued until euthanasia. (day +18). | No differences in clinical disability scores or body weight were identified between the groups over the 68 days following induction of EAE. |
| *Bernardes D et al 2016* | MOG-induced chronic EAE model. Female C57BL/6 mice were immunized with MOG_35-55_ peptide in CFA supplemented with *M.Tuberculosis*. N=47. | Forced exercise vs Sedentary  Forced exercise consisted of 30 minutes swimming per day, 5 days/week for 6 weeks with 7% body weight attached to tail. | EAE was induced following 5 weeks of training (day -35). | Exercised animals had significantly lower clinical scores from day 12 to day 38 dpi, at which point animals were sacrificed. |
| *Souza P*  *et al 2017* | MOG-induced chronic EAE model. Female C57BL/6 mice were immunized with MOG_35-55_ peptide in CFA supplemented with *M.Tuberculosis*. N=30 | Strength vs Endurance training  Strength training consisted of ladder climbing with a weight. Endurance training consisted for forced treadmill running. All animals were exercise 5 days/week for 4 weeks. | EAE was induced following 2 weeks of training (day -14). | Animals who underwent endurance training had a delay in onset of clinical signs and attenuated clinical severity scores compared with animals who performed strength training. |
| *Bernardes D et al 2017* | MOG-induced chronic EAE model. Female C57BL/6 mice were immunized with MOG_35-55_ peptide in CFA supplemented with *M.Tuberculosis*. N=24. | Forced exercise vs Sedentary  Forced exercise consisted of treadmill running at increasing speeds and inclines. | EAE was induced following 4 weeks of training (day -28). | No effect of training was found on clinical scores or walking speeds, but differences in gait were noted between exercised and sedentary animals. |
| *Einstein O*  *et all 2018* | Proteolipid protein (PLP)-induced transfer model. After stimulation with PLP, lymph node T cells were transferred from sedentary or trained donor SJL/JCrHsd mice into naïve recipients (also either sedentary or trained). | Forced exercise vs Sedentary in donor mice  Forced exercise consisted of treadmill running. | Animals were trained for 6 weeks before lymph-node T cell transfer (day-42). | Lymph-node T cell transfer from trained mice induced an attenuated clinical and pathological EAE in recipient mice compared with cells derived from sedentary animals. |
| *Bernardes D et al 2018* | MOG-induced chronic EAE model. Female C57BL/6 mice were immunized with MOG_35-55_ peptide in CFA supplemented with *M.Tuberculosis*. N=91. | Forced exercise vs Sedentary  Forced exercise consisted for daily treadmill running for 4-weeks.  Both trained and sedentary animals were administered DMF or GA following 1^st^ clinical relapse. | EAE was induced following 4 weeks of training (day -28). | No significant difference was observed between exercised and sedentary groups regarding clinical severity scores or body weight at initial relapse. However, trained animals had delayed and attenuated 2^nd^ relapses. In addition, differences were noted between sedentary and trained animals on DMT, suggesting exercise may modify effects of pharmacological treatment. |
| *Fainstein N*  *et al 2019* | Proteolipid protein (PLP)-induced transfer model. After stimulation with PLP, lymph node T cells were transferred from trained donor SJL/JCrHsd mice into naïve recipients. | Moderate vs High intensity training  Both consisted of treadmill training, of differing intensity | Animals were trained for 6 weeks before lymph-node T cell transfer (day -42). | Recipients from high-intensity trained donor mice had less severe EAE than recipients from moderate-intensity training donors. |
| *Xie Y et al*  *2019* | MOG-induced chronic EAE model. Female C57BL/6 mice were immunized with MOG_35-55_ peptide in CFA supplemented with *M.Tuberculosis*. | Moderate vs High intensity vs Sedentary  Moderate intensity training consisted of forced swimming. High intensity training consisted of forced swimming with 4% body weight. | EAE was induced following 6 weeks of training (day -42). | No significant difference in EAE clinical status was found between sedentary animals and those who underwent moderate intensity training. However, high intensity training was associated with attenuated EAE clinical scores, fewer infiltrating cells and less demyelination. |
| *Shahidi S et al 2020* | MOG-induced chronic EAE model. Female C57BL/6 mice were immunized with MOG_35-55_ peptide in CFA supplemented with *M.Tuberculosis*. N=96. | Forced exercise vs Voluntary vs Sedentary  Forced exercise consisted of swimming. Voluntary exercise consisted of free access of running wheel. | In one cohort, EAE was induced after 4 weeks of forced exercise/voluntary exercise/sedentary (day -28).  In a 2^nd^ cohort, exercise was initiated at onset of clinical signs (day 12). | Both modes of exercise (voluntary and forced) reduced clinical severity of EAE in mice, regardless of whether animals were trained before EAE induction or after. |
| *Torabimehr F et al 2020* | MOG-induced chronic EAE model. Female C57BL/6 mice were immunized with MOG_35-55_ peptide in CFA supplemented with *M.Tuberculosis*. N=40. | Forced exercise vs Voluntary vs Sedentary  Forced exercise consisted of swimming 5 days/week for 4 weeks. Voluntary exercise consisted of wheel access 1 hour/day, 5 days/week for 4 weeks. | EAE was induced following 4 weeks of training (day -28). | An increase in NCAM was seen in voluntary exercise animals (but not forced or sedentary). A reduction in NCAM has previously been reported to be associated with MS disease progression. |
| *Goldberg Y et al 2021* | Proteolipid protein (PLP)-induced transfer model. SJL/JCrHsd mice were trained and lymph-node T cells from trained donors were transferred to recipients. N=36. | High-intensity continual training (HICT) vs High-intensity interval training (HIIT). | Animals were trained for 5 weeks before lymph-node T cell transfer (day -35). | Attenuated EAE clinical severity was seen in recipient mice from both HICT and HIIT donors. HIIT altered T cell profiles, whereas HCT reduced overall T cell proliferation. |
| *Zaychik Y et al 2021* | Proteolipid protein (PLP)-induced transfer model. PLP_139-151_ lymph node transfer of T-cells from donor mice into trained or sedentary SJL/JCrHsd recipient mice. N=20. | Forced exercise vs Sedentary  Forced exercise consisted of HICT on treadmill. | Animals were trained for 6 weeks before lymph-node T cell transfer (day -42). | Both groups developed clinical signs with 7-10 days of transfer and were followed up for 50 days. T-cell transfer into HICT trained recipients resulted in a milder EAE compared with T-cell transfer into Sedentary mice. |
| *Hamdi L et al 2022* | Proteolipid protein (PLP)-induced transfer model. Lymph-node T cells from trained or sedentary female SJL/JCrHsd donors were transferred to trained or sedentary recipients. | Forced exercise vs Sedentary, both in donors and recipients  Forced exercise consisted of 6 weeks HICT treadmill training. | Animals were trained for 6 weeks before lymph-node transfer (day -42). | T-cell transfer from HICT donors resulted in attenuated EAE severity in recipients. T-cell transfer from sedentary donors into HICT trained recipients also resulted in a milder EAE. |
| *Nazari M et al 2022* | MOG-induced chronic EAE model. Female C57BL/6 mice were immunized with MOG_35-55_ peptide in CFA supplemented with *M.Tuberculosis*. N=24. | Forced exercise vs Sedentary  Forced exercise consisted of 30 minutes swimming, 5 days/week, for 4 weeks. | EAE was induced following 4 weeks of training (day -28). | In exercise animals, fetuin-A levels were reduced to slightly above control. |
| *Gilio L et al 2022* | MOG-induced chronic EAE model. Female C57BL/6 mice were immunized with MOG35-55 peptide in CFA supplemented with *M.Tuberculosis*. | Voluntary exercise vs Sedentary  Voluntary exercise consisted of a wheel-equipped cage. | Wheel access initiated 1 day after immunization (day 1). | Exercised animals shower reduced depressive behaviors in the pre-symptomatic phase of EAE compared with sedentary animals. |
| *El-Eman M et al 2022* | Male, Sprague-Dawley rats were immunized with spinal cord homogenate in CFA supplemented with *M.Tuberculosis*. N=36 | Forced exercise vs Sedentary  Forced exercise consisted of 30/minutes per day on treadmill.  A proportion of both trained and sedentary animals were also treated with Mitoxantone. | EAE was induced following 2 weeks of training (day -14). | Training alone did not alter EAE severity scores. Unexpectedly, in animals treated with Mitoxantone, the exercise groups had worse EAE clinical scores than the sedentary group. |

Data from these studies were used to create Figure 1. For systematic search strategy see Table 1. MOG = myelin oligodendrocyte glycoprotein; PLP = proteolipid protein; EAE = experiemental autoimmune encephalomyelitis; CFS = complete Freund’s adjuvant; dpi = days post injection; NCAM = neural cell adhesion molecule.
