# Supplementary material for: Multiple sclerosis and exercise—A disease-modifying intervention of mice or men?
Source: Front Neurol. 2023 Oct 10;14:1190208. doi: 10.3389/fneur.2023.1190208 (PMC10598461; doi:10.3389/fneur.2023.1190208)
Supplement: Supplementary file 2 [file Table_2.docx]

**Supplementary Table 2** outlining 11 systematic reviews and meta-analyses of exercise intervention studies in pwMS and disability and disease durations of the cohorts included.

| Review/  Meta-analysis | Aim | Number of studies included | Number of participants included | EDSS range and average across studies | Disease duration range and average across studies |
| --- | --- | --- | --- | --- | --- |
| *Studies assessing the effect of exercise on immune markers* | | | | | |
| *Shoberi P et al 2022* | Comparison of pre and post exercise BDNF levels | N=13  Aerobic exercise=6  Anaerobic exercise=2  Combined training=5 | N=271 | Range 1.5–6.5  Mean 3.1 (SD 0.8) | Range 4.4-16.6 years  Mean 9.1 (SD 7.2) years |
| *Najafi P et al 2022* | Cytokine profiling following exercise in pwMS | N=22  Mix of endurance, aerobic, swimming, Pilates, resistance and combination training | N=742  419 exercise  323 controls | Range 0-8  Mean 3.1 (SD 0.2) | Disease duration not documented. Cohorts included RRMS, PPMS and SPMS. |
| *Studies assessing the effects of exercise on aerobic fitness* | | | | | |
| *Langeskov-Christensen et al 2015* | Assess the training effects on VO2 max in pwMS | N=17  Aerobic exercise=17 | N=214  125 exercise  89 controls | Range 0-8  Mean 2.9 (SD 1.1) | Disease duration not documented. Cohorts included RRMS, PPMS and SPMS. |
| *Studies assessing the effect of exercise on participation and cognition* | | | | | |
| *Edwards T et al 2022* | How does exercise training improve participation in pwMS | N=23  Aerobic exercise=13  Resistance=4  Mixed=6 | N=1093  558 exercise  535 control | Range 1.5-8  Mean 4 (SD 0.9) | Range 2.7-22.3 years  Mean 10 (SD 6.2) years |
| *Gharakhanlou R et ak 2021* | Exercise training and cognitive performance in pwMS | N=13  Aerobic=8  Resistance=1  Combination=5 | N=639 | Mean 3.4 | Disease duration not documented. Cohorts included RRMS, PPMS and SPMS. |
| *Studies assessing the effect of exercise on Quality of life and general health perception* | | | | | |
| *Andreu-Caravaca L et al 2022* | Effects and optimal dosage of resistance training on strength, functional capacity, general health perception and fatigue in pwMS | N=44 | N=1105 | Range 0-7.5  Mean 3.4 (0.8) | Range 1-28 years  Mean 10.7 (SD 4) years |
| *Reina-Gutierrez S et al 2022* | The type of exercise most beneficial of quality of life in pwMS | N=45  Sensorimotor=27  Aerobic=15  Combination=11  Mind-body=8  Resistance=4 | N=2428 | Mean 3.5 (SD 1.5) | Range 2.6-18.7 years  Mean 11 (SD 6.7) years |
| *Latimer-Cheung A et al 2013* | Effects of exercise on fitness, mobility, fatigue and quality of life in pwMS | N=54  Mix of aerobic, resistance and mixed training | N=1318 | Mean 3.7 (SD 1.4) | Mean 11 (SD 4.5) years |
| *Studies assessing the effects of exercise on upper limb function* | | | | | |
| *Neira V et al 2022* | Effects of exercise on upper limb function for pwMS | N=8 | N=215 | Range 3.5-9  Mean 5.4 (SD 11) | Range 9-27 years  Mean 18.1 (SD 9.3) years |
| *Studies assessing the effects of exercise on lower limb function* | | | | | |
| *Taul-Madson L et al 2021* | Aerobic versus resistance training for improving lower limb function and fatigue in pwMS | N=27  Aerobic=16  Resistance=11 | N=966 | Range 1.5-7  Mean 3.1 (1.2) | Range 2.7-18.6 years  Mean 8.8 years |
| *Studies assessing the effects of exercise on fatigue* | | | | | |
| *Heine M et al 2016* | Cochrane review of the effects of exercise therapy on fatigue | N=36 | N=1603 | Range 2-6.3  Mean 3.9 (SD 1.2) | Range 6-16.1 years  Mean 9.2 (SD 6.1) years |

Data from these studies were used to create Figures 1 and 2. For systematic search strategy see Table 1. EDSS = expanded disability severity score; BDNF = brain derived neurotrophic factor; RRMS = relapsing remitting multiple sclerosis; PPMS = primary progressive multiple sclerosis; SPMS = secondary progressive multiple sclerosis.
